# Spontaneous splenic rupture: a case report and literature review

**DOI:** 10.3389/fsurg.2025.1687868

**Published:** 2026-01-05

**Authors:** Xinxing Liu, Tingliang Fu, Donghua Li, Lei Geng, Shuai Sun

**Affiliations:** 1 Department of Pediatric Surgery, Binzhou Medical University Hospital, Binzhou, Shandong, China; 2 Department of Anesthesiology, Binzhou Medical University Hospital, Binzhou, Shandong, China

**Keywords:** atraumatic splenic rupture, pediatric surgery, splenic rupture, spontaneous splenic rupture, surgery

## Abstract

Spontaneous splenic rupture in children is a rare condition with limited documented cases in the medical literature. It can occur in both enlarged and normal-sized spleens. Clinical manifestations may include abdominal pain, splenomegaly, diminished or absent bowel sounds, Kehr's sign, Ballance's sign, and abdominal guarding. Imaging studies (computed tomography and ultrasound) serve as crucial diagnostic tools for splenic rupture. Splenectomy is no longer considered standard treatment due to the associated risk of overwhelming post-splenectomy infection (OPSI). Current therapeutic approaches prioritize hemostasis and spleen preservation. Prognosis depends on timely diagnosis and adequate management. Therefore, clinicians must maintain high vigilance for patients presenting with unexplained acute abdominal pain accompanied by hemodynamic instability. This article reports a pediatric case of spontaneous splenic rupture and reviews the literature to summarize the pathophysiology, clinical features, and management strategies for this condition.

## 1 Introduction

Splenic rupture is an uncommon condition. Given the spleen's highly vascular nature, splenic hemorrhage carries significant mortality, particularly when diagnosis is delayed—potentially leading to catastrophic outcomes (1). Spontaneous splenic rupture can be classified into traumatic and atraumatic types. Splenic hemorrhage is most commonly caused by blunt injury, while the incidence of atraumatic splenic rupture is significantly lower (2). Atraumatic splenic rupture, also known as spontaneous splenic rupture (SSR), is a rare but potentially life-threatening condition typically associated with underlying pathological conditions, including neoplasms, hematologic disorders, and inflammatory processes. These pathological states induce splenomegaly and tissue friability, creating the foundation for rupture (1–4). Splenic rupture may still occur in normal spleens, though its incidence is relatively low. Since it is unrelated to any underlying pathology, this condition is termed idiopathic splenic rupture (5, 6).

Given the spleen's critical role in host defense, the primary therapeutic objectives are hemostasis and splenic preservation. However, when patients develop hemodynamic instability accompanied by uncontrolled rupture or recurrent splenic hemorrhage, surgical intervention should be considered the first-line treatment (7, 8). Early identification through diagnostic tools such as computed tomography (CT) and ultrasonography is crucial for improving patient outcomes. The presence of hemoperitoneum is highly suggestive of the diagnosis, and imaging modalities like abdominal CT or ultrasound further aid in confirmation (9–11).

We report a case of SSR in a child. In light of the paramount importance of timely diagnosis and appropriate surgical management in such cases, we will focus on discussing the diagnostic challenges and required surgical approaches for managing SSR. Aiming to describe the clinical characteristics of pediatric splenic rupture, summarize treatment approaches and outcomes, and provide a literature review on this rare condition.

## 2 Case report

A 6-year-old female patient visited our hospital due to sudden abdominal pain for 1 day. The patient has no history of trauma. Upon admission, the child appeared lethargic. Vital signs include: blood pressure 110/75 mmHg, axillary temperature 36.7 °C, heart rate 106 beats per minute, respiratory rate 26 breaths per minute, and weight 20 kg. Physical examination revealed deep tenderness in the right upper quadrant and right periumbilical area of the abdomen. The abdominal muscles were slightly tense, but there was no rebound tenderness. Laboratory findings showed a Complete Blood Count (CBC) with a White Blood Cell (WBC) count of 6.2 ×  10⁹/L, a Red Blood Cell (RBC) count of 3.9 × 10¹²/L, Hemoglobin (Hb) of 114 g/L, Platelets (Plt) of 227 × 10⁹/L, Hematocrit of 35%, Neutrophils at 66.1%, and Lymphocytes at 25.7%. Color Doppler Ultrasound demonstrated no abnormalities in the liver, gallbladder, pancreas, spleen, kidneys, mesenteric vessels, bowel, bilateral ovaries, or uterus; however, an anechoic fluid collection was visualized within the abdominal cavity, with a maximum anteroposterior diameter of approximately 6.4 cm.

Following hospitalization, the decision was made to perform an ultrasound-guided abdominal paracentesis for diagnostic analysis of the fluid. Approximately 20 mL of non-clotting blood was drained. Despite rapid fluid resuscitation, the patient remained hemodynamically unstable. The rechecked blood pressure was 96/60 mmHg. Repeat CBC revealed a RBC count of 2.9 × 10¹²/L, Hb of 89 g/L, and hematocrit of 25%. Given the critical clinical deterioration and absence of a definitive diagnosis, an emergency diagnostic laparoscopy was performed.

An arc-shaped incision was made above the umbilicus to establish a pneumoperitoneum. Upon entering the abdominal cavity with a laparoscope, diffuse hemoperitoneum with significant pelvic blood accumulation, totaling approximately 350 mL. Two 5 mm tissue grasping forceps were inserted at the left subcostal midclavicular line and 2 cm to the right of the umbilicus to assist in exploration. A hematoma was identified between the spleen and liver in the left upper quadrant, anterior to the gastrosplenic ligament. After evacuation of the hematoma, a 2 cm longitudinal laceration was visualized on the superior pole of the spleen near the visceral peritoneal surface, with active pulsatile bleeding. Hemostasis was achieved by packing absorbable hemostatic gauze and applying local omental compression. No rebleeding was observed during a 30 min monitoring period. The incision was then closed. Estimated intraoperative blood loss from the laceration was 250 mL. Transfusion included 100 mL of Fresh Frozen Plasma (FFP) and 3 units of Packed Red Blood Cells (PRBCs). Postoperative repeat CBC showed: RBC count 3.2  ×  10¹²/L, Hb 98 g/L, hematocrit 28%. The patient was given tranexamic acid injection (100 mg intravenously, b.i.d.) for 5 days and azithromycin (200 mg intravenously, q.d.) for 3 days. The patient maintained hemodynamic stability with no evidence of rebleeding. Pre-discharge CBC revealed: RBC 3.6  ×  10¹²/L, Hb 101 g/L, hematocrit 31%.

This pediatric patient underwent a two year follow-up, remaining clinically well and asymptomatic throughout the period.

## 3 Discussion

Spontaneous rupture of a histologically normal spleen is an exceedingly rare phenomenon, distinct from traumatic splenic rupture, which involves identifiable physical injury (12, 13). This entity occurs without discernible precipitating factors or underlying pathology, posing significant diagnostic and therapeutic challenges that frequently lead to delayed recognition and management.

The pathophysiological mechanisms underlying spontaneous rupture of histologically normal spleens remain elusive. Several theoretical frameworks have been proposed (13–15): Congenital or acquired vascular abnormalities may predispose the spleen to spontaneous rupture. Microaneurysms or arteriovenous malformations can compromise the integrity of the splenic parenchyma. In susceptible individuals, minor and often unrecognized microtrauma from activities such as heavy lifting or violent coughing may contribute to splenic rupture. Conditions causing abrupt elevation of intra-abdominal pressure—including emesis or strenuous exertion—can precipitate rupture in otherwise healthy spleens. Cases without an identifiable etiology are classified as idiopathic SSR.

SSR predominantly occurs in patients with underlying disorders, particularly hematologic malignancies, infectious diseases, or systemic inflammatory processes (2–4, 13). Etiologic pathogens include but are not limited to Epstein–Barr virus (EBV), hepatitis viruses, Salmonella typhi, Rickettsia prowazekii (typhus), bacterial endocarditis pathogens, Mycobacterium tuberculosis, Brucella spp., Treponema pallidum (syphilis), and cytomegalovirus (CMV) (2, 3, 7, 14). Notably, infectious mononucleosis (IM) secondary to EBV represents the most prevalent infectious cause of SSR (16).

Timely diagnosis of SSR is pivotal for successful management. Diagnostic challenges necessitate integration of physical examination, laboratory findings, and imaging studies. Vital signs may reveal tachycardia and hypotension secondary to hypovolemic shock. Physical signs include: abdominal tenderness, splenomegaly, diminished or absent bowel sounds, Kehr's sign, Ballance's sign, and abdominal guarding (involuntary muscle rigidity) (2, 17–21). Abdominal pain most frequently localizes to the left upper quadrant, though it may manifest in any abdominal quadrant. The pain exhibits variable characterization, ranging from sharp/stabbing to cramping/colicky in nature (1, 15, 21). Kehr's sign manifests as severe discomfort in the scapular or acromial region—predominantly the left shoulder—elicited by the passive leg-raising maneuver in supine position, resulting from diaphragmatic irritation by intraperitoneal blood or other irritants (19). Ballance's sign is characterized by dullness on percussion over the left flank (particularly the left costal region) due to perisplenic hematoma accumulation, with concomitant tympany in the right anterior abdominal wall (20). The absence of trauma history often leads to misdiagnosis as other acute conditions, including acute appendicitis, gastrointestinal perforation, ruptured diverticular abscess, ectopic pregnancy, aortic dissection, myocardial infarction, or pulmonary embolism (13, 22). Laboratory studies typically reveal anemia, characterized by an acute hemoglobin drop of >20 g/L within 24 h (14).

Imaging studies constitute a critical modality for diagnosing splenic rupture, with contrast-enhanced CT (CECT) serving as the preferred initial investigation (10, 11, 13, 14, 21). CECT differentiates etiological factors such as pancreatic body/tail enlargement and splenic flexure tumors, and reveals critical findings—including massive free intraperitoneal fluid and isolated subcapsular hematomas—that directly inform surgical or nonoperative management decisions. Liu et al. (10) recommend CECT for all hemodynamically stable patients with suspected SSR. However, given its portability, rapid acquisition, high sensitivity for solid organ injuries and hemoperitoneum, and absence of ionizing radiation, ultrasonography (US) remains the first-line imaging modality for pediatric populations or hemodynamically unstable patients (13, 23, 24). Furthermore, for unexplained peritoneal effusion, abdominal paracentesis is a quick, convenient, and safe diagnostic method to rapidly clarify the nature of the fluid (25, 26). In this case, the initial US indicated the presence of abdominal effusion without suggesting injury to abdominal organs, leading to the decision to perform an abdominal puncture first to clarify the nature of the effusion. The puncture revealed non-coagulable blood, and due to the child's hemodynamic instability, CECT was not chosen; instead, an emergency laparoscopic exploration was performed.

Magnetic resonance imaging (MRI) has been investigated as a diagnostic tool for visceral injuries, including splenic rupture. It demonstrates exceptional sensitivity for detecting minimal intraparenchymal hemorrhage, subtle capsular tears, and associated soft-tissue injuries. However, its utility in acute settings is constrained by prolonged acquisition times, stringent patient stability requirements, and reduced accessibility compared to CT. Consequently, MRI is typically reserved for follow-up evaluations or specific clinical scenarios necessitating advanced tissue characterization capabilities (13, 14).

The management spectrum for SSR encompasses conservative observation with intensive monitoring, bed rest, splenic artery embolization, splenorrhaphy (including techniques such as Gelfoam packing, omental patching, capsular suturing, or absorbable mesh application), and splenectomy (5, 8, 13, 27). Treatment selection hinges upon the patient's hemodynamic stability and severity of splenic injury, typically graded using the American Association for the Surgery of Trauma (AAST) scale (28). Injuries graded I-III are classified as mild to moderate and are typically amenable to nonoperative management. Conversely, grade IV-V injuries represent high-grade lesions carrying significant mortality risk and generally necessitate surgical intervention. In our case, the child had a splenic capsular tear with a depth of <1 cm, corresponding to a Grade I injury according to the AAST classification, but presented with hemodynamic instability. Therefore, hemostasis was achieved using absorbable hemostatic gauze packing. In our case, the child had a splenic capsular tear with a depth of <1 cm, corresponding to a Grade I injury according to the AAST classification, but presented with hemodynamic instability. Therefore, hemostasis was achieved using absorbable hemostatic gauze packing.

Recent studies advocate nonoperative management as first-line therapy for splenic rupture hemorrhage to mitigate overwhelming post-splenectomy infection (OPSI) risk, particularly critical in pediatric patients who face substantially higher post-splenectomy sepsis vulnerability (5, 8, 27–30). Surgical decision-making should be expedited in hemodynamically unstable patients. When operative management is indicated, spleen-preserving techniques—including surgical suturing combined with absorbable gelatin sponges, mesh, or tissue adhesives—should be prioritized to retain immunologic function (27, 30). When the above treatment plans fail to control bleeding, splenectomy should be performed without delay (22, 31). Overwhelming post-splenectomy infection (OPSI or PSS) is a complication following splenectomy, most commonly caused by Gram-positive encapsulated bacteria. OPSI is primarily caused by Streptococcus pneumoniae, with less common pathogens including Haemophilus influenzae, Neisseria meningitidis, and Gram-negative bacilli. Currently, there are highly effective strategies to prevent infections in post-splenectomy patients, including vaccination, prophylactic antibiotic use (which should be continued for 3 years in children and adolescents), and immediate initiation of antibiotic treatment upon suspected infection. Patients should be educated about the nature and risks of PSS and seek immediate medical attention when ill or febrile. Through these measures and precautions, the risk of PSS can be significantly reduced or even completely avoided (32, 33).

The prognosis of SSR hinges on the timeliness of diagnosis and therapeutic adequacy. Prompt intervention is critical for outcome optimization (8, 13, 27). Therefore, clinicians should enhance their recognition of this condition.

## 4 Conclusion

Although spontaneous rupture of a histologically normal spleen is rare, it constitutes a critical emergency. Its underlying pathophysiology remains incompletely elucidated, mandating further research. Clinicians must maintain a high index of suspicion for this entity in patients presenting with unexplained acute abdominal pain and hemodynamic instability to ensure expeditious diagnosis and intervention.

## Data Availability

The original contributions presented in the study are included in the article/Supplementary Material, further inquiries can be directed to the corresponding authors.
